# Supplementary material for: Chromothripsis during telomere crisis is independent of NHEJ, and consistent with a replicative origin
Source: Genome Res. 2019 May;29(5):737–49. doi: 10.1101/gr.240705.118 (PMC6499312; doi:10.1101/gr.240705.118)
Supplement: Supplemental Material [file supp_gr.240705.118_Supplemental_file_1.zip › contigs/annotated_contigs/DB56/contig.2.DB56_length_670_mean_cov_10.1388059701.docx]

**DB56_length_670_mean_cov_10.1388059701**

```
TGTTATTAGTATATCTACAAGAGATTAAACTGTGATAACTTAATTTTTTTTTTTTTTTTTTTTACAATTTTGGTAAAATGAGAGAGTGT
 >chr18:68913463-68913770 + E=2e-134
TTATTATTATTATTATTATTATTATTATTATTATTATTTGAGACAGCATCTTGCTCTGTCACACACGCTGGAGTGCAGTGGCATGATCA

TAGCCCATGGCAACCTTGACCTCCTGGGCTCAAGCAATCCTTCACCTCAGCCTCTTAAGTAACTGGGACCAGAGGCACATGCCATTACG

CCTGGCTAATTTTTAAATTATTTGTGTAG|AC|CTATTATATATAATTTTTTATGCTACATATTTATATCCTATATATGTGATATATAT
                              >chr18:67818440-67818814 + E=3e-207
ATGATTATATAAAGTTATCATCAATACAGCTATGTACCTAAAATACAATTACTTTGAACCAACACTTAGAGTAACTATCAGGAATTTTT

TTCTCAATATGTGATAGATAATAGTATTTTGTTAGCAGCTATTTAAGGTGAGAGGGGAAAAAAAGCTACTTGAAATATTAATGGAATTT

AAATACAATATAGAAGGTAAAAATACAATAAATAATGAGTTACATGAATACTACTTTTTTTCATTCTGCAAATTTCTACTTCCTATTAT

CAACAATTCAAAGGTCAAAAAGTTACACACATACACTCACATATGTATT
```
